# Dynamic trajectory of cerebral autoregulation recovery after carotid artery stenting in severe carotid stenosis

**DOI:** 10.3389/fneur.2026.1735460

**Published:** 2026-02-06

**Authors:** Xiaojuan Wang, Bo Li, Rong Guo, Xuke Zhang, Mingrui Zhu, Jiaxin Liu, Xiangnan Li, Muhui Lin

**Affiliations:** 1 Department of Neurology, Dalian Medical University, Dalian, China; 2 Department of Neurology, The People’s Hospital of Liaoning Province, Shenyang, China; 3 Department of Clinical Neurophysiology, The People’s Hospital of Liaoning Province, Shenyang, China; 4 Department of Neurology, Bengbu Medical College, Anhui, China

**Keywords:** carotid artery stenting, dynamic cerebral autoregulation, internal carotid artery, surgery, time course

## Abstract

**Objective:**

To investigate the temporal changes and recovery patterns of dynamic cerebral autoregulation (dCA) after carotid artery stenting (CAS) in patients with severe internal carotid artery stenosis.

**Methods:**

In this prospective study, 44 patients undergoing CAS (19 symptomatic and 25 asymptomatic) and 44 age-matched healthy controls were enrolled. Patients in the CAS group underwent dCA assessments at four time points: pre-CAS (baseline, within 24 h prior to surgery), postoperative day 1 (POD 1), postoperative day 3 (POD 3), and 1 month postoperatively (POM 1). dCA was quantified using transfer function analysis (TFA), including phase difference (PD) and gain, to evaluate dCA.

**Results:**

Pre-CAS, patients had bilateral dCA impairment, worse on the affected (stenotic) side (PD: affected side: 30.56 ± 19.87° vs. unaffected side: 43.29 ± 23.29°, *p* < 0.001; both lower than healthy controls at 52.96 ± 14.82°, affected side: *p* < 0.001, unaffected side: *p* = 0.019). After CAS, dCA recovered rapidly. The affected side’s PD improved to the unaffected (non-stenotic) level by day 1 (POD 1 PD: affected side: 36.94 ± 20.59°, unaffected side: 41.69 ± 23.29°, *p* > 0.05), and both sides reached the level of healthy controls by day 3 (POD 3 PD: affected side: 47.71 ± 23.64°, unaffected side: 51.07 ± 24.43°, *p* > 0.05). The recovery trajectory was consistent between symptomatic and asymptomatic subgroups and aligned with the overall patient cohort.

**Conclusion:**

Unilateral severe carotid stenosis impairs bilateral dCA, while CAS significantly restores cerebral autoregulation. Recovery is faster on the affected side, reaching the unaffected level within 1 day, and both hemispheres reach healthy control levels by day 3. Early CAS intervention can timely improve dCA regardless of symptom status.

## 1 Introduction

Carotid artery stenosis is a major contributor to ischemic stroke, accounting for 15–20% of cases (1, 2). Progressive narrowing not only heightens the risk of ischemia but also impairs dynamic cerebral autoregulation (dCA), the brain’s ability to stabilize cerebral blood flow (CBF) despite fluctuations in arterial pressure (2–5). In patients managed with medical therapy alone, approximately 26% of stenotic arteries progress to complete occlusion within 2 years of follow-up, markedly increasing stroke risk (6). Revascularization strategies, such as carotid artery stenting (CAS) or carotid endarterectomy (CEA), effectively restore cerebral hemodynamics and reduce the incidence of ischemic events (7–9).

dCA is central to cerebrovascular homeostasis. By adjusting arteriolar tone, it buffers changes in cerebral perfusion pressure (CPP), thereby limiting the impact on CBF and preventing hypoperfusion or hyperperfusion injury (10, 11). Evidence indicates that dCA is impaired in carotid stenosis and that impairment correlates with stenosis severity (3, 12). This dysfunction may predispose patients to perioperative hyperperfusion or acute ischemic complications (13–15). While prior studies suggest that CAS enhances dCA and that recovery after CEA is delayed but evident by 1 month (7, 8), the longitudinal course of dCA after CAS remains poorly defined.

The lack of clarity regarding postoperative dCA dynamics constrains blood pressure management and risk prediction. To address this gap, we systematically examined dCA at multiple time points before and after CAS. In addition, we compared recovery trajectories between the stenotic and contralateral hemispheres and between symptomatic and asymptomatic patients, aiming to delineate subgroup-specific patterns of dCA restoration.

## 2 Methods

### 2.1 Participants and study design

This prospective study was approved by the Ethics Committee of Liaoning Provincial People’s Hospital (approval number: 201728). All participants provided written informed consent and could withdraw at any time.

Between December 2024 and July 2025, patients scheduled for CAS were consecutively recruited. Inclusion criteria were: 1. Unilateral internal carotid artery stenosis of 70–99% confirmed by carotid ultrasound (Delica, Shenzhen, China) and validated with computed tomographic angiography (CTA) or digital subtraction angiography (DSA); 2. Eligible for CAS and willing to undergo the procedure; 3. Adequate bilateral temporal bone windows for Transcranial Doppler (TCD; MultiDop X2, DWL, Sipplingen, Germany) monitoring, with clear waveforms; 4. Signed informed consent. Exclusion criteria included: 1. Need for emergent CAS, or evidence of cerebral infarction with hemorrhagic transformation, or intracranial vascular malformations/aneurysms; 2. Contralateral carotid artery or middle cerebral artery (MCA) stenosis ≥70% or occlusion, as MCA stenosis ≥70% is considered hemodynamically significant and may influence cerebral autoregulation assessment (2); 3. Conditions potentially affecting dCA assessment (e.g., anxiety, depression, impaired consciousness, agitation); 4. Poor temporal window or difficulty securing TCD probe; 5. Life expectancy <1 month or inability to complete the study; 6. Poor compliance with follow-up or treatment; 7. dCA data with coherence < 0.14.

An age-matched healthy control group without moderate or severe carotid stenosis was recruited for comparison. To ensure data comparability, exclusion criteria were applied based on items 3, 4, and 7 above.

Baseline clinical data, including age, sex, smoke, drank, hypertension, diabetes, and dyslipidemia, were collected. dCA measurements in the CAS group were performed at four time points: pre-CAS (baseline, within 24 h prior to surgery), postoperative day 1 (POD1), postoperative day 3 (POD 3), and 1 month postoperatively (POM 1). Controls underwent a single assessment.

### 2.2 dCA assessment

Measurements were performed in a quiet room (22–24 °C) after 10–15 min of rest. Continuous arterial blood pressure was recorded using a servo-controlled volume-clamp device (Finometer Model 1, FMS, Amsterdam, Netherlands) with the finger cuff at heart level.

TCD with 2 MHz probes was used through bilateral temporal windows to measure middle cerebral artery blood flow velocity at 45–60 mm depth. End-tidal carbon dioxide (EtCO_2_) was monitored using a near-infrared spectroscopy-based capnograph. Once stable waveforms were obtained, probes were fixed in a head frame to maintain consistent position and depth. Following calibration, continuous data were recorded for ≥10 min and stored for analysis. All measurements were performed by a single operator. To assess the intra-rater reliability of the TCD-based measurements, a random subset of 10 participants underwent a repeat assessment by the same operator within a 1-week interval. The intraclass correlation coefficient (ICC) for the key dCA parameter, phase difference in the very low frequency band, was calculated using a two-way random-effects model for absolute agreement. The ICC was 0.87 (95% CI: 0.82–0.91), indicating excellent reliability.

### 2.3 dCA data analysis

Stable 10-min segments of arterial blood pressure (ABP) and cerebral blood flow velocity (CBFV) signals were analyzed using transfer function analysis (TFA). Parameters calculated included phase, gain, and coherence across very low frequency (VLF, 0.02–0.07 Hz), low frequency (LF, 0.07–0.20 Hz), and high frequency (HF, 0.20–0.50 Hz) bands (16, 17). Bilateral results were averaged.

Phase reflects the temporal shift between ABP (input) and CBFV (output), gain indicates damping, higher phase and lower gain indicate better dCA function, with phase considered the most reliable metric (3, 18). Coherence was used to evaluate signal reliability as a key metric for assessing the outcome of transfer function analysis. Guided by the methodological standards proposed in the white paper of the International Cerebral Autoregulation Research Network (CARNet), a coherence threshold >0.14 was adopted as the statistical criterion for data inclusion: this threshold corresponded to approximately 13 effective data segments (degrees of freedom) obtained from 10-min signals processed using standardized spectral analysis (window length 102.4 s, 50% overlap) and indicated statistical significance at the *p* < 0.05 level. Data with coherence <0.14 were excluded (16, 17).

VLF band primarily reflects myogenic activity and slow sympathetic regulation, and demonstrates heightened sensitivity in chronic cerebrovascular pathologies such as carotid artery disease and stroke. Therefore, the analysis focused primarily on VLF parameters (16, 19).

### 2.4 Statistical analysis

Statistical analyses were conducted using IBM SPSS Statistics (27.0 SPSS; IBM Corp, West Grove, PA, USA). Normality was assessed with the Kolmogorov–Smirnov test. Normally distributed data are reported as mean ± SD and compared with independent *t*-tests; non-normal data are presented as median (interquartile range, IQR) and compared with the Mann–Whitney *U* test. Categorical variables are expressed as percentages and compared using *χ*^2^ test.

Repeated-measures ANOVA (RM-ANOVA) was applied to compare dCA parameters across time points and between affected and unaffected sides, with time and side as repeated factors. Paired *t*-tests with Bonferroni correction were used for side-by-side comparisons at each time point. RM-ANOVA was also employed to examine differences between symptomatic and asymptomatic patients over time. A *p*-value < 0.05 was considered statistically significant. The ‘interaction effect’ in repeated-measures ANOVA was used to examine whether the effect of one factor (e.g., time) on the outcome variable was dependent on another factor (e.g., affected/unaffected side or symptomatic grouping). A significant interaction (*p* < 0.05) indicated that the effects of the two factors were not independent, meaning that the pattern of one factor’s influence differed across the levels of the other factor.

## 3 Results

**Figure 1:**
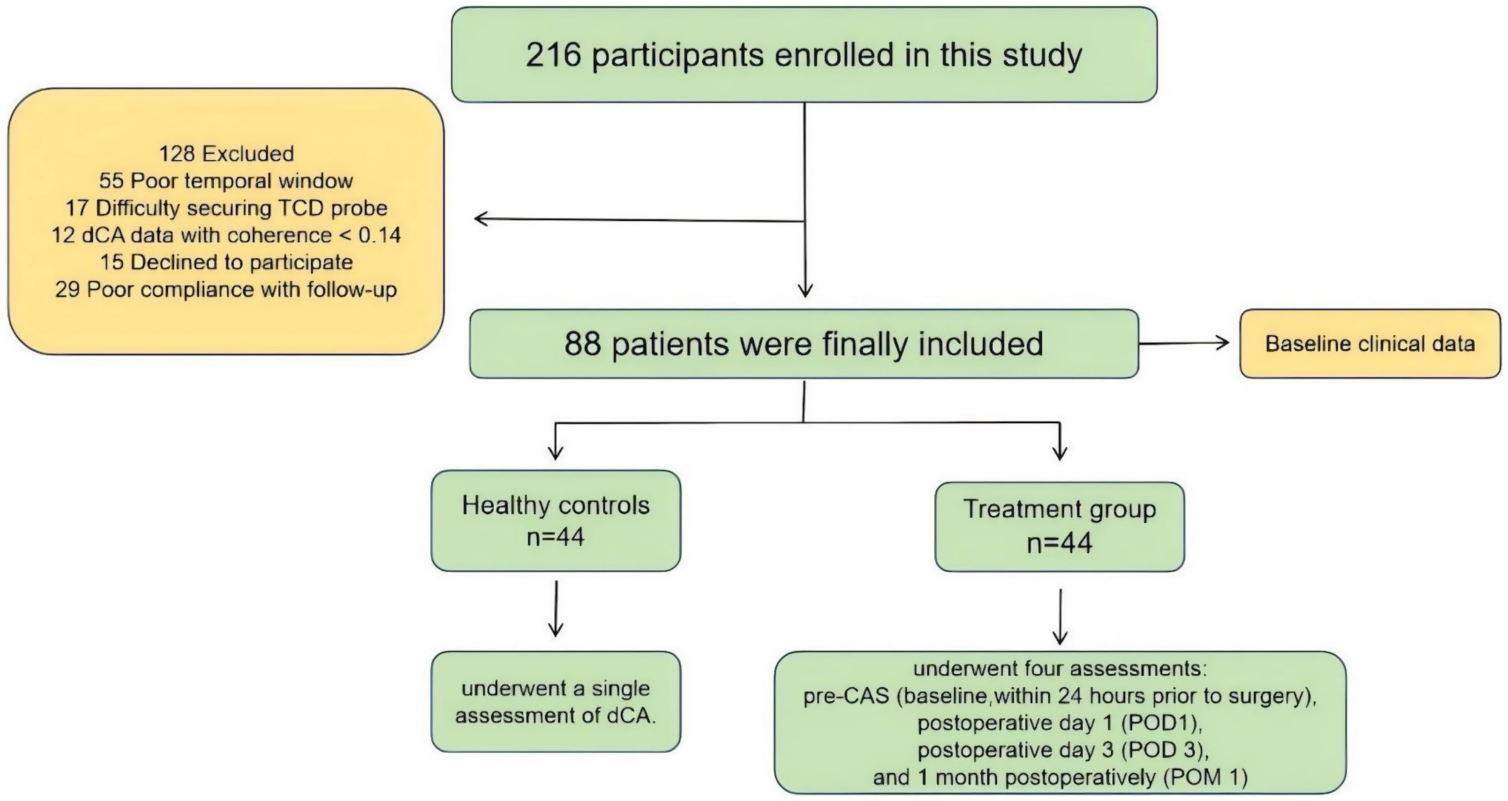
Flow diagram of included studies. N, number.

After rigorous screening, as detailed in the flowchart (Figure 1), a total of 44 patients who met the inclusion criteria for CAS were enrolled and completed dCA monitoring at four time points. The CAS group had a mean age of 65.34 ± 7.47 years and included 42 men. Among them, 19 patients experienced hemispheric ischemic events within the past 30 days (6 transient ischemic attacks [TIA] and 13 ischemic strokes), classified as symptomatic stenosis, while the remaining 25 patients had asymptomatic stenosis.

Following CAS, one patient exhibited hyperperfusion on ultrasound without clinical symptoms, and no new ischemic events occurred during the 1-month follow-up.

For comparison, 44 age-matched healthy controls (mean age 64.95 ± 7.92 years, all male) were included. Participant characteristics are summarized in Table 1.

**Table 1:** Clinical characteristics of participants.

Variables	Total	Symptomatic	Asymptomatic	Controls	*P*_1_	*P*_2_
	*N* = 44	*N* = 19	*N* = 25	*N* = 44		
Age,y	65.34 ± 7.47	62.68 ± 8.62	67.36 ± 5.86	64.95 ± 7.92	0.814	0.130
Gender (male)	42 (95.5%)	18 (94.7%)	24 (96.0%)	44 (100%)	0.494	0.247
Smoke	27 (61.4%)	13 (68.4%)	14 (56.0%)	23 (52.3%)	0.389	0.492
Drink	27 (61.4%)	13 (68.4%)	14 (56.0%)	22 (50.0%)	0.283	0.401
Hypertension	26 (59.1%)	9 (47.4%)	17 (68.0%)	24 (54.5%)	0.667	0.357
Diabetes	20 (45.5%)	6 (31.6%)	14 (56.0%)	15 (34.1%)	0.276	0.144
Dyslipidemia	5 (11.4%)	3 (15.8%)	2 (8.0%)	10 (22.7%)	0.156	0.346
Previous stroke	15 (34.1%)	4 (21.1%)	11 (44.0%)	11 (25.0%)	0.350	0.165
Previous heartdisease	7 (15.9%)	3 (15.8%)	4 (16.0%)	5 (11.4%)	0.534	0.776

Data are presented as *n* (%) or mean ± SD. *P*_1_ values are for differences between total patients and controls. *P*_2_ values are for differences among symptomatic stenosis patients, asymptomatic stenosis patients, and controls.

Effect size: Standardized Mean Difference (SMD) was calculated to quantify the magnitude of between-group differences for key baseline variables between the total patient group and healthy controls. For continuous variables (e.g., Age), Cohen‘s d was used; for binary variables, the Phi coefficient was used. SMD values are presented with 95% confidence intervals (CI). Conventionally, |SMD| < 0.2 indicates a negligible difference, 0.2–0.5 a small difference, 0.5–0.8 a moderate difference, and ≥0.8 a large difference. The SMD for Age was 0.05 (95% CI: −0.37 to 0.47).

Table 2 presents the repeated-measures summary of dCA parameters across different sides (affected vs. unaffected) and time points. The data indicate that PD changed over time, with distinct temporal trends observed between the affected and unaffected sides.

**Table 2:** Summary of repeated measurements for different parameters of dCA across different sides and time points.

Parameter	Time		Different sides		Interaction	
	*F*	*P*	*F*	*P*	*F*	*P*
Phase difference (degree)	17.741	0.001***	2.296	0.133	1.613	0.195
Gain (%/mmHg)	9.892	<0.001***	4.740	0.032*	4.167	0.027*

dCA, dynamic cerebral autoregulation. The interaction (Side × Time) tested whether the trajectories of dCA parameters over time were consistent between the affected and unaffected sides. A significant interaction (*p* < 0.05) indicates different recovery patterns between sides, meaning the effect of time depends on the side (affected vs. unaffected).

“*” denotes *P* < 0.05. “**” denotes *p* ≤ 0.01. “***” denotes *p* ≤ 0.001.

### 3.1 CAS-related temporal changes in dCA parameters

**Figure 2:**
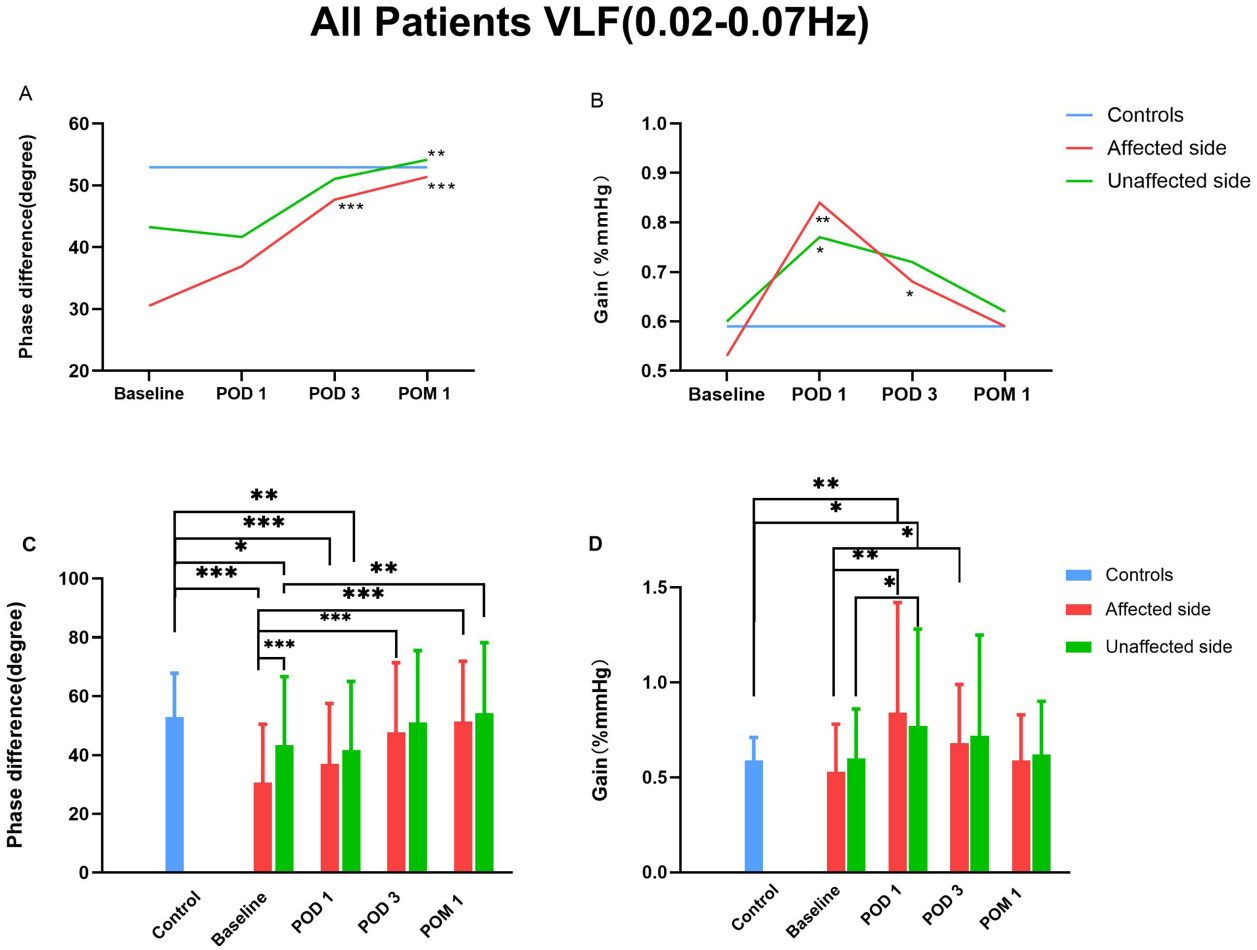
Time course of dCA before and after CAS. CAS, carotid artery stenting; dCA, dynamic cerebral autoregulation. “*” denotes *p* < 0.05. ** denotes *p* < 0.01. “***” denotes *p* < 0.001.

The temporal changes of dCA parameters are summarized in Table 3 and Figure 2. Compared with healthy controls, patients undergoing CAS exhibited significantly lower PD on both the affected and unaffected sides before CAS and on POD 1 (*p* < 0.05). On POD 3, PD values on both sides showed no significant difference from controls.

**Table 3:** Time course of dCA before and after CAS.

Total (*N* = 44)	Patients with CAS						Controls (*N* = 44)
	Baseline (*N* = 44)	POD1 (*N* = 44)	POD 3 (*N* = 44)	POM 1 (*N* = 44)	*F*	*P*	
**Phase difference (degree)**	**52.96 ± 14.82**
Affected side	30.56 ± 19.87^ab^	36.94 ± 20.59^b^	47.71 ± 23.64^c^	51.41 ± 20.42^c^	15.52	<0.001***	
Unaffected side	43.29 ± 23.29^b^	41.69 ± 23.29^b^	51.07±24.43	54.17 ± 24.01^c^	4.939	0.006**
**Gain (%mmHg)**	**0.59 ± 0.12**
Affected side	0.53 ± 0.25	0.84 ± 0.58^bc^	0.68±0.31^c^	0.59 ± 0.24	8.135	0.001***	
Unaffected side	0.60 ± 0.26	0.77±0.51^bc^	0.72 ± 0.53	0.62 ± 0.28	3.324	0.041*
**Coherence**	**0.64 (0.049)**
Affected side	0.65 (0.098)	0.64 (0.050)	0.66 (0.078)	0.66 (0.068)	1.177	0.319	
Unaffected side	0.64 (0.055)	0.65 (0.040)	0.66 (0.075)	0.66 (0.078)	1.162	0.327

CAS, carotid artery stenting; dCA, dynamic cerebral autoregulation.

“*” denotes *P* < 0.05. “**” denotes *P* ≤ 0.01. “***” denotes *P* ≤ 0.001.

^a^*P* < 0.05 compared with unaffected side.

^b^*P* < 0.05 compared with controls.

^c^*P* < 0.05 compared with baseline.

Repeated measures analysis revealed significant temporal changes in PD (affected side: *F* = 15.520, *p* < 0.001; unaffected side: *F* = 4.939, *p* = 0.006). Relative to baseline, PD of affected side increased significantly at POD 3 (47.71 ± 23.64° vs. 30.56 ± 19.87°, *p* = 0.001) and further at POM 1 (51.41 ± 20.42° vs. 30.56 ± 19.87°, *p* < 0.001). PD of unaffected side was significantly higher at 1 month compared with baseline (54.17 ± 24.01° vs. 43.29 ± 23.29°, *p* = 0.002).

Regarding gain values, on the affected side, gain increased significantly at POD 1 compared with baseline (0.84 ± 0.58 vs. 0.53 ± 0.25, *p* = 0.005), on the unaffected side, gain increased significantly at POD 1 compared with baseline (0.77 ± 0.51 vs. 0.60 ± 0.26, *p* = 0.025), followed by a gradual decrease over subsequent time points, indicating a trend toward recovery.

### 3.2 Comparison of dCA between the affected and unaffected sides

The dCA parameters for the affected and unaffected sides are summarized in Table 3. At baseline, the PD on the affected side was significantly lower than that on the unaffected side (30.56 ± 19.87° vs. 43.29 ± 23.29°, *p* < 0.001). No significant differences in PD were observed between the two sides at POD 1 (36.94 ± 20.59° vs. 41.69 ± 23.29°), POD 3 (47.71 ± 23.64° vs. 51.07 ± 24.43°), or POM 1 (51.41 ± 20.42° vs. 54.17 ± 24.01°) (all *p* > 0.05).

Gain values at all four time points also did not differ significantly between the affected and unaffected sides (all *p* > 0.05).

### 3.3 Dynamic cerebral autoregulation across time in symptomatic and asymptomatic stenosis patients

Repeated measures of dCA parameters for both hemispheres across different time points in symptomatic and asymptomatic stenosis patients are summarized in Table 4. Statistical analysis showed significant differences in PD between groups (affected side: *F* = 16.845, *P* = <0.001; unaffected side: *F* = 4.893, *p* = 0.007), and no significant group-by-time interaction was observed (affected side: *F* = 2.028, *p* = 0.113; unaffected side: *F* = 1.057, *p* = 0.358). Statistical analysis showed significant differences in Gain between groups (affected side: *F* = 9.823, *P* = <0.001; unaffected side: *F* = 3.585, *p* = 0.033), and a significant group-by-time interaction was observed at affected side (*F* = 3.398, *p* = 0.049); there is no significant group-by-time interaction was observed at affected side at unaffected side (*F* = 1.193, *p* = 0.308).

**Table 4:** Summary of repeated measurements for different parameters of dCA across time points and patients in different groups (symptomatic stenosis patients and asymptomatic stenosis patients).

Parameter	Time		Different groups		Interaction	
	*F*	*P*	*F*	*P*	*F*	*P*
**Phase difference (degree)**						
Affected side	16.845	<0.001***	0.154	0.696	2.028	0.113
Unaffected side	4.893	0.007**	1.224	0.271	1.057	0.358
**Gain (%/mmHg)**						
Affected side	9.823	<0.001***	0.004**	0.949	3.398	0.049*
Unaffected side	3.585	0.033*	0.001***	0.972	1.193	0.308

dCA, dynamic cerebral autoregulation. The interaction (Group × Time) tested whether the trajectories of dCA parameters over time were consistent between symptomatic and asymptomatic patients. A significant interaction (*P* < 0.05) indicates different recovery patterns between groups, meaning the effect of time depends on symptomatic status.

“*” denotes *P* < 0.05. “**” denotes *p* ≤ 0.01. “***” denotes *p* ≤ 0.001.

Further separate analysis of symptomatic and asymptomatic stenosis patients revealed that the changes in PD over time were similar in both groups for affected and unaffected hemispheres (Table 5), consistent with the results for the entire cohort. In both groups, affected PD increased by POD 3 compared with healthy controls.

**Table 5:** Time course of dCA before and after CAS between symptomatic stenosis patients and asymptomatic stenosis patients.

Total (*N* = 44)	Baseline (*N* = 44)	POD 1 (*N* = 44)	POD 3 (*N* = 44)	POM 1 (*N* = 44)	*F*	*P*	Controls (*N* = 44)
**Phase difference (degree)**	**52.96 ± 14.82**
**Symptomatic (*N* = 19)**							
Affected side	23.61 ± 13.61^ab^	37.39 ± 19.90^bc^	49.31 ± 28.32^c^	51.95 ± 23.90^c^	12.609	<0.001***	
Unaffected side	35.61 ± 14.42^b^	41.62 ± 22.61^b^	48.74 ± 22.85	50.69 ± 23.26^c^	4.632	0.015*
**Asymptomatic (*N* = 25)**							
Affected side	35.84 ± 22.38^ab^	36.60 ± 21.50^b^	46.48 ± 19.90	51.01 ± 17.85^c^	5.407	0.005*	
Unaffected side	49.14 ± 25.64	41.74 ± 24.26^b^	52.85 ± 25.88	56.82 ± 24.69	2.465	0.097
**Gain (%/mmHg)**	**0.59 ± 0.12**
**Symptomatic (*N* = 19)**							
Affected side	0.42 ± 0.14^ab^	0.97 ± 0.62^bc^	0.67 ± 0.27^c^	0.59 ± 0.22c	8.939	0.003**	
Unaffected side	0.53 ± 0.21	0.83 ± 0.39b^c^	0.70 ± 0.28^c^	0.66 ± 0.30	8.373	0.001***
**Asymptomatic (*N* = 25)**							
Affected side	0.62 ± 0.28	0.75 ± 0.53	0.69 ± 0.34	0.58 ± 0.25	1.693	0.201	
Unaffected side	0.65 ± 0.29	0.73 ± 0.59	0.74 ± 0.66	0.59 ± 0.27	1.226	0.309
**Coherence**	**0.64 (0.049)**
**Symptomatic (*N* = 19)**							
Affected side	0.66 (0.120)	0.65 (0.060)	0.67 (0.110)	0.67 (0.110)	1.464	0.235	
Unaffected side	0.64 (0.050)	0.65 (0.060)	0.66 (0.040)	0.68 (0.070)	0.931	0.309
**Asymptomatic (*N* = 25)**							
Affected side	0.65 (0.105)	0.63 (0.050)	0.66 (0.070)	0.66 (0.060)	0.83	0.482	
Unaffected side	0.63 (0.065)	0.65 (0.040)	0.66 (0.085)	0.66 (0.100)	0.325	0.807

CAS, carotid artery stenting; dCA, dynamic cerebral autoregulation.

“*” denotes *P* < 0.05. “**” denotes *P* ≤ 0.01. “***” denotes *P* ≤ 0.001.

^a^*P* < 0.05 compared with unaffected side.

^b^*P* < 0.05 compared with controls.

^c^*P* < 0.05 compared with baseline.

### 3.4 Dynamic cerebral autoregulation in symptomatic and asymptomatic stenosis patients

**Figure 3:**
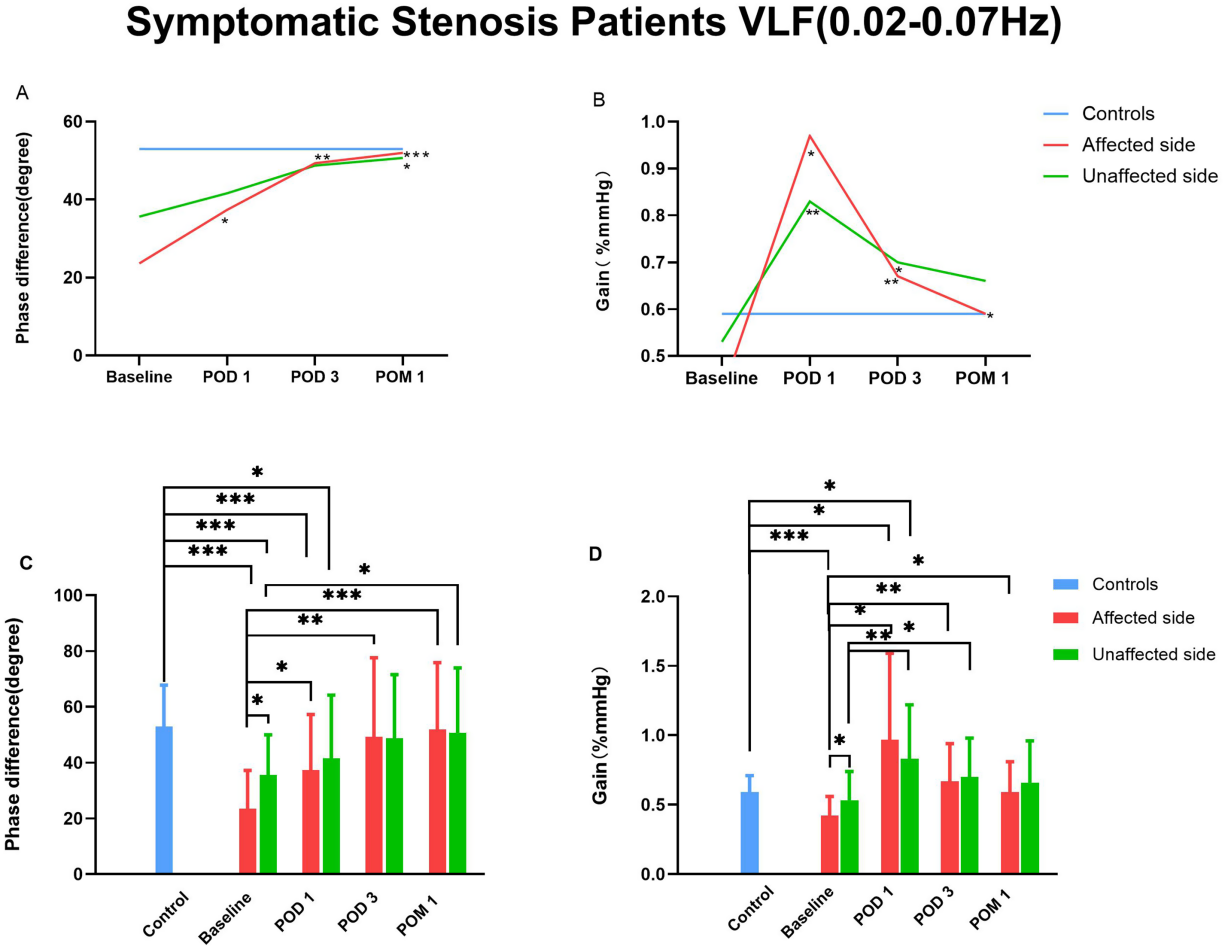
Time course of dCA of symptomatic stenosis patients before and after CAS. CAS, carotid artery stenting; dCA, dynamic cerebral autoregulation. “*” denotes *p* < 0.05.”**” denotes *p* < 0.01. “***” denotes *p* < 0.001.

**Figure 4:**
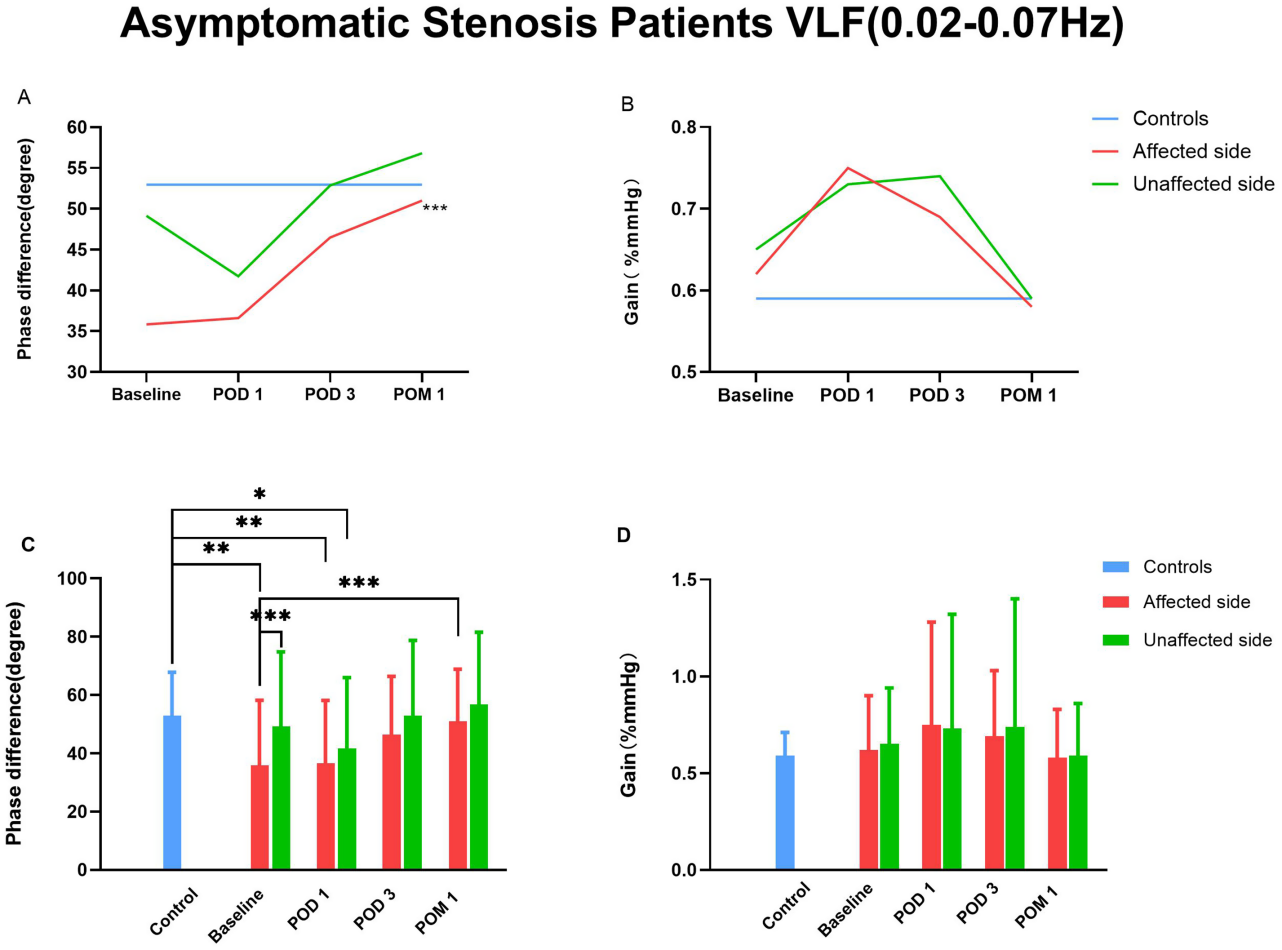
Time Course of dCA of Asymptomatic Stenosis Patients Before and After CAS. CAS, carotid artery stenting; dCA, dynamic cerebral autoregulation. “*” denotes *p* < 0.05. “**” denotes *p* < 0.01. “***” denotes *p* < 0.001.

The comparison of dCA parameters between symptomatic and asymptomatic stenosis patients is summarized in Table 5, Figures 3 and 4. Repeated-measures analysis revealed significant changes in affected PD across time points for both groups (symptomatic: *F* = 12.609, *p* < 0.001; asymptomatic: *F* = 5.407, *p* = 0.005). Compared with baseline, affected PD in symptomatic patients increased markedly on POD 1 whereas in asymptomatic patients, the increase was more pronounced at POM 1.

At all time points, the PD on the affected side was significantly lower than that on the unaffected side (Symptomatic: 23.61 ± 13.61° vs. 35.61 ± 14.42°, *p* = 0.018; Asymptomatic: 35.84 ± 22.38° vs. 49.14 ± 25.64°, *p* = 0.001). No significant differences in PD were observed between the two sides at POD 1, POD 3 or POM 1 (all *p* > 0.05).

Compared with healthy controls, the PD on the affected and unaffected sides for symptomatic stenosis patients showed significant differences (affected: 23.61 ± 13.61° vs. 52.96 ± 14.82°, *P* < 0.001; unaffected: 35.61 ± 14.42° vs. 52.96 ± 14.82°, *P* < 0.001). No significant differences in PD were observed between the two sides at POD 3. However, for asymptomatic stenosis patients, there is only a significant difference in PD on the affected side (35.84 ± 22.38° vs. 52.96 ± 14.82°, *p* = 0.002), no significant differences in PD were observed between the two sides at POD 3.

## 4 Discussion

This study demonstrated a gradual improvement of dCA in patients following CAS. dCA of affected side recovered to the level of the contralateral side by POD 1, while bilateral dCA showed significant improvement by POD 3, approaching values observed in healthy controls. Compared with baseline, PD of affected side significantly increased at POD 3 and PD of unaffected side at POM 1, indicating a time-dependent recovery of dCA. Further analysis revealed that although preoperative dCA in the unaffected side of patients with asymptomatic stenosis remained unimpaired compared to healthy controls, no significant differences were observed in postoperative recovery between symptomatic and asymptomatic stenosis patients, suggesting that preoperative symptom status had limited impact on short-term dCA restoration.

Previous studies have reported that impaired dCA in carotid stenosis can reduce cerebral blood volume and flow velocity, resulting in hemodynamic instability (HI), which may compromise distal cerebral perfusion and increase the risk of clinically apparent ischemia. CAS-related microembolus clearance may be affected, leading to a higher incidence of new ischemic lesions detected on diffusion-weighted-imaging (DWI) (20). dCA impairment has been identified as a key hemodynamic biomarker for acute, asymptomatic ischemic lesions (ASIL) after CAS, with predictive accuracy enhanced when combined with dyslipidemia and plaque morphology (19). In the present study, no neurological deficits were observed during 1-month follow-up; however, cranial DWI was not performed, and the occurrence of new asymptomatic ischemic lesions could not be determined. Notably, severe impairment of dCA before or in the early phase after the procedure may exacerbate perioperative cerebral hemodynamic fluctuations and instability, thereby altering local flow patterns and endothelial shear stress, which could potentially act as a contributing factor to in-stent thrombosis (19, 21). However, this hypothetical relationship warrants further validation through future studies incorporating high-resolution vascular imaging and systematic postoperative hemodynamic monitoring.

Compared with CEA, CAS appeared to yielded a faster recovery of dCA. Previous research has shown that CEA causes more severe endothelial injury, with dCA improvement observed only after approximately 1 month (8, 22). This interpretation is based on indirect comparisons with historical data and requires prospective randomized controlled trials for verification. In contrast, our CAS cohort exhibited PD increases within several days postoperatively, suggesting relatively less endothelial damage and more rapid functional restoration. All patients had varying degrees of preoperative dCA impairment, yet no clinically significant hyperperfusion syndrome occurred postoperatively, which may be attributable to strict blood pressure management (about 120/80 mmHg) (23, 24). One patient demonstrated ultrasonographic hyperperfusion without clinical symptoms; dCA in this patient returned to normal by 1 month, highlighting a potential link between blood pressure control and early dCA stabilization.

Furthermore, gain of affected side was elevated on POD 1 but gradually decreased over time, whereas phase values had already returned to unaffected levels. This aligns with previous findings that phase is a more sensitive indicator of dCA changes than gain (8, 9, 13). The transient increase in gain on POD 1 may reflect early instability of dCA, with both hemispheres requiring adjustment to a new equilibrium. Therefore, close monitoring of blood pressure and hemodynamics during the early postoperative period, in combination with interventions such as remote ischemic preconditioning, may facilitate dCA recovery (9, 25).

Our study confirms that CAS rapidly restores dCA function in patients with severe carotid stenosis, irrespective of symptomatic status, providing hemodynamic support for early revascularization as a cerebroprotective strategy. However, long-term outcomes depend not only on restored flow, but also on intraprocedural embolic protection and long-term implant biocompatibility. Recent evidence indicates that optimized stent design (e.g., micro-mesh) significantly reduces perioperative embolic load and silent cerebral infarction risk (26, 27), suggesting synergy between device innovation and hemodynamic management. Future studies should examine how different stent types—particularly those with enhanced embolic protection—affect dCA recovery, especially in high-risk plaque patients.

Furthermore, interventional devices are evolving from mechanical scaffolding toward biological healing. Bioresorbable stents may degrade after vascular reconstruction, potentially avoiding long-term risks of permanent implants, while drug-eluting devices locally deliver agents to suppress neointimal hyperplasia (28). dCA could serve as a key indicator for evaluating whether such novel devices better preserve cerebrovascular autoregulation. Integrating hemodynamic monitoring with imaging may advance an integrated “structure–function-healing” strategy for optimizing carotid therapy.

Overall, the results indicate that CAS can improve dCA function within a short period postoperatively, Early postoperative dCA dynamics suggest that timely hemodynamic management may be crucial for protecting cerebral autoregulation and optimizing long-term outcomes.

Despite providing insights into the time-dependent recovery of dCA following CAS, this study has several limitations. First, only patients eligible for TCD monitoring were included, which led to the exclusion of individuals with inadequate temporal bone windows. Previous studies have reported that approximately 20.7% of Asian individuals have at least one inadequate temporal bone window (7), potentially introducing selection bias and limiting the generalizability of the findings. Second, the study population was predominantly male, likely reflecting the lower prevalence of carotid stenosis in females (29, 30); this gender imbalance may have influenced the results, highlighting the need for further clinical studies to validate these findings and mitigate potential sex-related biases. Finally, the sample size was relatively small, the follow-up period was limited to 1 month, and imaging assessments were not performed, leaving the long-term trajectory of dCA recovery and the occurrence of asymptomatic ischemic lesions uncertain. Future studies with larger cohorts, extended follow-up, and combined imaging evaluations are warranted to confirm these results and to explore strategies for optimizing early dCA recovery.

## 5 Conclusion

Based on our findings, CAS leads to a rapid short-term improvement in bilateral dCA function, with the ipsilateral side recovering to the level of the contralateral side by POD 1, and both sides approaching levels observed in healthy controls by day 3. These results highlight the critical role of early hemodynamic management in protecting cerebral autoregulation, providing new evidence to support neuroprotection following revascularization.

## Data Availability

The raw data supporting the conclusions of this article will be made available by the authors, without undue reservation.
